# Insight into Population Structure and Evolutionary Analysis of the Emerging Tomato Brown Rugose Fruit Virus

**DOI:** 10.3390/plants11233279

**Published:** 2022-11-28

**Authors:** Ali Çelik, Sevgi Coşkan, Ali Ferhan Morca, Adyatma Irawan Santosa, Davoud Koolivand

**Affiliations:** 1 Department of Plant Protection, Faculty of Agriculture, Bolu Abant İzzet Baysal University, Bolu 14030, Turkey; 2 Directorate of Central Plant Protection Research Institute, Gayret Mah. Fatih Sultan Mehmet Bulv., Yenimahalle, Ankara 06172, Turkey; 3 Department of Plant Protection, Faculty of Agriculture, Universitas Gadjah Mada, Jl. Flora No. 1, Sleman, Yogyakarta 55281, Indonesia; 4 Department of Plant Protection, Faculty of Agriculture, University of Zanjan, Zanjan 45371, Iran

**Keywords:** divergence time, evolutionary constraints, genetic variation, phylogenetic analysis

## Abstract

A total of 112 symptomatic tomatoes (*Solanum lycopersicum* L.) and 83 symptomatic pepper (*Capsicum* spp.) samples were collected in Ankara, Eskişehir, Bartın, and Zonguldak provinces of Turkey during 2020–2021. Six tomatoes and one pepper sample (3.6%) tested positive for tomato brown rugose fruit virus (ToBRFV, genus *Tobamovirus*) infection by DAS-ELISA and RT-PCR. ToBRFV-positive tomato and pepper plants were removed from greenhouses as soon as possible, and the greenhouses and tools were disinfected completely. Phylogenetic analysis on the complete CP sequences suggested the clustering of 178 GenBank isolates and 7 novel isolates into three groups. A study using DnaSP software showed very low genetic variation among current global ToBRFV isolates. All four ORFs of the virus genome were under strong negative evolutionary constraints, with a ω value range of 0.0869–0.2066. However, three neutrality tests indicated that most populations of the newly identified ToBRFV are currently expanding by assigning statistically significant negative values to them. The very low *F*_ST_ values (0.25 or less) obtained by all comparisons of the isolates from Europe, the Middle East, China, and America concluded that there is no clear genetic separation among currently known isolates from different geographic origins. The divergence time of ToBRFV was estimated to be in the middle of the course of the evolution of 11 tested tobamoviruses. The time to the most recent common ancestors (TMRCAs) of ToBRFV were calculated to be 0.8 and 1.87 with the genetically closest members of *Tobamovirus*. The results of this study could improve our understanding on the population structure of the emerging ToBRFV.

## 1. Introduction

Tomato (*Solanum lycopersicum* L.) is one of the most significant and widely produced horticulture crops in the world. Like other cultivated vegetables, tomato is exposed to different types of viruses, which often lead to changes not only in local crop management practice but also wider quarantine policy, particularly when unexpected and sudden outbreaks occur. Tomato brown rugose fruit virus (ToBRFV) has been one of such viruses that have caused the most devastating epidemic diseases on tomato crops globally in recent years [1]. The virus was also known to infect and induce yield losses on pepper (*Capsicum* spp., family Solanaceae) at a lesser degree [2,3].

ToBRFV is a member of the genus *Tobamovirus*, which, unlike other members of the family *Virgaviridae*, contains only a single genomic RNA [4]. The nearly 6400 nt single-stranded positive-sense RNA (+ssRNA) genome of ToBRFV has four open reading frames (ORFs) that express the following: two replication-related protein complexes of ca. 126 and 183 kDa by ORF1a and ORF1b, respectively (the second protein is synthesised by partial stop codon suppression); the movement protein (MP) of ca. 30 kDa by ORF2; and the 17.5 kDa coat protein (CP) encoded by 30-coterminal sub-genomic RNAs by ORF3 [5].

ToBRFV infection causes symptoms such as interveinal discolouration, distortion, and mosaics on leaves, deformation and necrosis on young leaves, necrosis and deformation in sepals, and browning, deformation, embossing, and necrosis in young fruits [6]. Typical of tobamoviruses, physical contact with infected crops is enough to mechanically transmit this virus to healthy plants [5]. It can also be dispersed over long distances via contaminated seeds, infected fruits, and other plant parts. Even worse, due to its highly stable properties, a ToBRFV particle can stay in a greenhouse long after post-harvesting, on equipment, and other surfaces, including clothing, wires, plastic, concrete, and soil [7,8].

It is no wonder that the easily transmissible ToBRFV has been found in many countries since its relatively recent first detection in Jordan [5] and in Israel on tomatoes bearing the resistance gene *Tm-2^2^* [9]. Up to now, ToBRFV infection has been reported in Mexico [2], the United States of America (California) [10], Germany [11], Italy [12], Palestine [13], Turkey [14], the United Kingdom [15], China [16], Greece [17], Holland [18], Spain [19], Iran [20,21], and France [22]. Despite stricter quarantine measures, the virus spread is likely still ongoing due to ToBRFV’s capacity to travel via imported fruits and seeds; thus, these reports maybe understated, and the virus is becoming a worldwide concern in the future.

Because of their huge population size, quasispecies, the absence of proofreading systems for genomes, and rapid generation rates, allowing for genetic heterogeneity, RNA viruses have a high potential to develop and adapt swiftly under natural selective pressures [23]. The high frequency of mutation, recombination, and reassortment in virus genomes promotes the development of novel forms, which quickly spread across the viral population when the genetic variation leads in a functional gain [24]. Therefore, understanding the population structure of a virus and examining the diversity are very important in terms of pathogen control. 

The genome of ToBRFV isolates from different regions was known to be closely linked, implying that they descended from a single common origin [7]. However, research relevant to the population of ToBRFV is limited. A study and genomic comparison of several tobamoviruses by [25] suggested that a host-shifting occurrence of the ToBRFV variants happened with a comparatively low mutation rate in a very short period. Phylogenetic analyses have also been conducted, but no general agreement on isolates clustering was achieved [8,26,27,28,29]. Therefore, the genome sequences of all isolates available in NCBI GenBank were retrieved and analysed, together with the coat protein region of the novel Turkish isolates reported in this study, to provide the first insight into the global population structure of the emerging ToBRFV.

## 2. Results

### 2.1. Incidence and Sequencing

**Figure 1:**
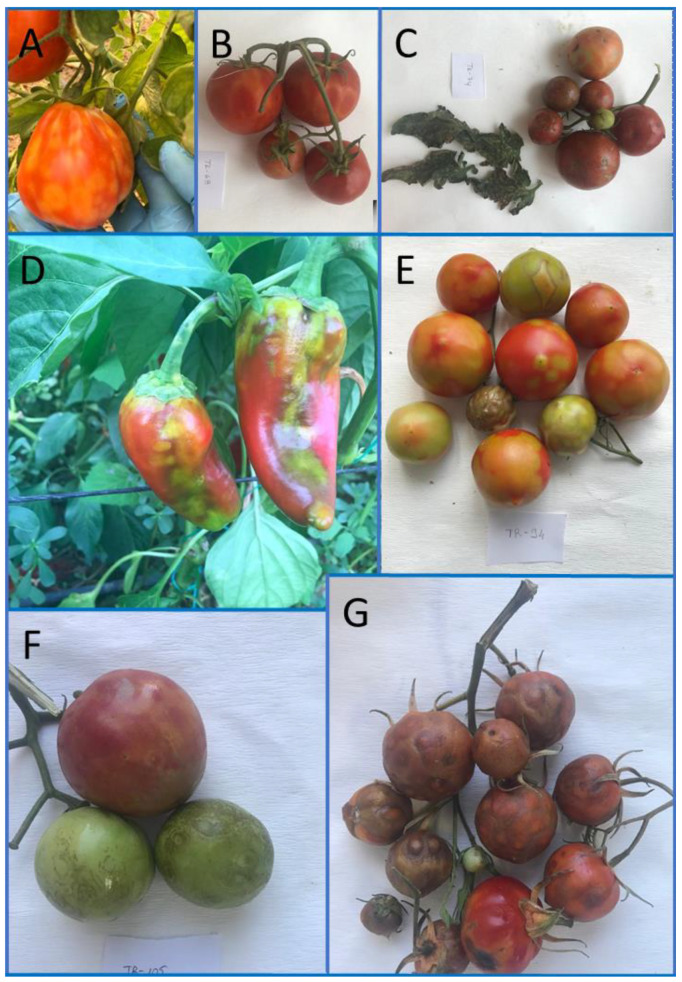
Different symptoms on plants infected by ToBRFV Turkish isolates. (**A**) Spots on fruit and leaf mosaic on tomato infected by TR-49; (**B**) spots on tomato fruits infected by TR-68; (**C**) leaf and fruit malformation on tomatoes infected by TR-74; (**D**) yellow spots and severe malformation on peppers infected by TR-79; (**E**) spots and blisters on tomato fruits infected by TR-94; (**F**) spots on tomato fruits infected by TR-105; and (**G**) severe blisters on tomato fruits infected by TR-108.

Upon testing by DAS-ELISA and RT-PCR, only seven samples: six tomatoes (A 4, E 1, and B 1) and one pepper (A 1) were positive for ToBRFV infection, giving a low disease incidence of 3.6%. All infected plants showed severe symptoms, including leaf mosaic and spots and blisters on fruits (Figure 1).

The targeted genome regions of seven isolates were sequenced and NCBI GenBank accession codes were provided for them (Table S1). The recovered 824 bp region (201 bp partial MP, 480 bp complete CP, and 143 bp 3`-UTR) encodes 274 amino acids (aa) and is homologous to nt 5514-6337 of the complete genome sequence of the ToBRFV reference isolate NC_028478.

### 2.2. Recombination and Phylogenetic Analyses

RDP4 scan on the complete genome sequences alignment of 174 isolates did not detect any recombination event. Evidence of recombination was also not found on ORF4/CP alignment of 185 isolates, which included 4 other GenBank isolates and 7 novel isolates.

**Figure 2:**
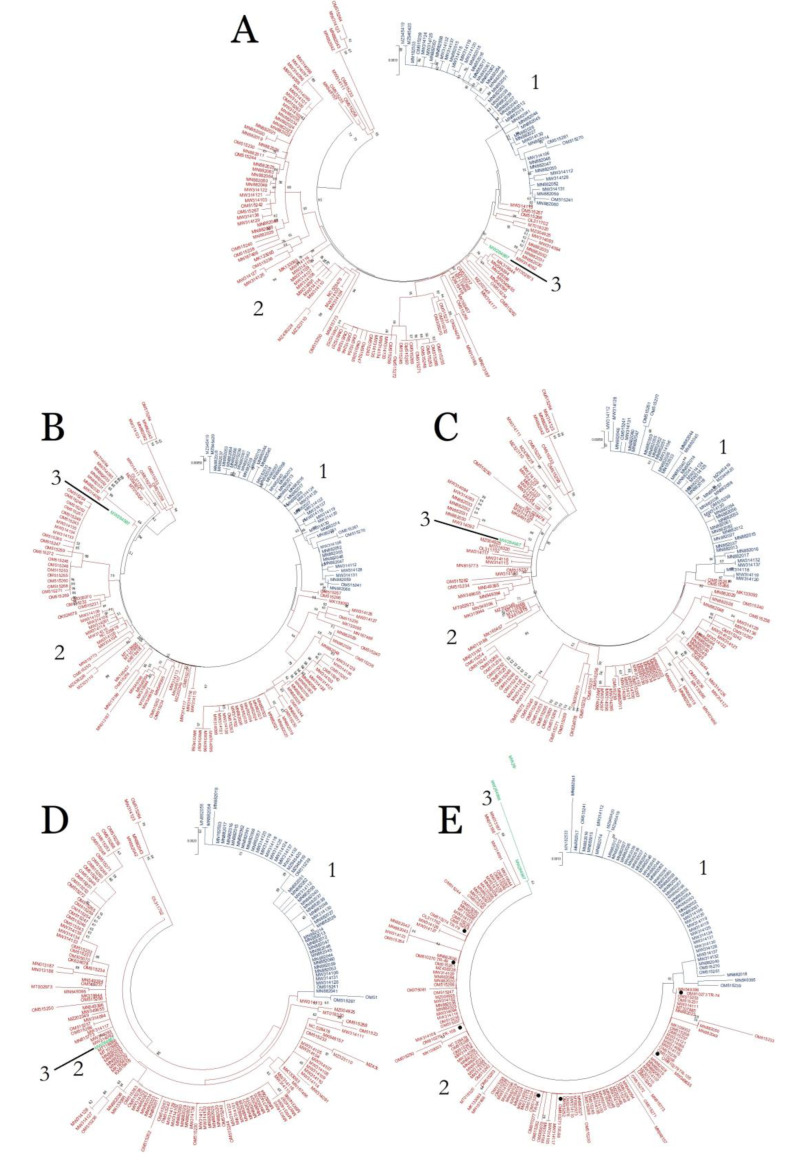
Neighbor-Joining phylogenetic analysis using Tamura 3-parameter model (TN92) with uniform rates among sites (1000 bootstrap replicates, only >50% values were shown) by MEGA X software to show the distribution of 174 isolates in each phylogenetic tree based on (**A**) the complete genome, (**B**) ORF1, (**C**) ORF2, (**D**) ORF3 (MP), and 185 isolates for (**E**) ORF4 (CP). The three phylogrouping systems (1: Blue; 2: Red; and 3: Green) were based on CP tree. Black dots are seven Turkish isolates identified in the present study.

Phylogenetic analysis of the ORF4/CP alignment of 185 isolates by MEGA X suggested that ToBRFV phylogeny can be divided into three groups (Figure 2). The vast majority of the isolates were distributed into groups 1 and 2, with 134 and 49 isolates, respectively. Two French isolates: 22006291-H (MW284987) and 22006291-L (MW284988) were positioned in group 3 (Figure 2).

Further analysis on the complete genome and the other three ORF alignments deduced that the clustering of the 49 isolates in group 1 was fully intact in the complete genome, ORF1, ORF2, and ORF3 phylogenetic trees, and showed very close relation to these isolates (Figure 2). On the other hand, 134 isolates in group 2 were dispersed into different groups in the complete genome, ORF1, ORF2, and ORF3 phylogenetic trees (Figure 2).

### 2.3. Genetic Variation and Selection Pressure Analyses

Analysis by DnaSP provided quantitative estimation of low genetic variation among current global ToBRFV isolates. All tested populations obtained very low π (<0.1) and k values. CP was shown to be the most conserved region, demonstrated by the lowest Hd, *S*, η, *k*, and π values among analysed ORFs which were all obtained by the gene. In addition to this, analysis on the CP sequences of 185 isolates found only 34 distinct haplotypes (h) (Table 1).

**Figure 3:**
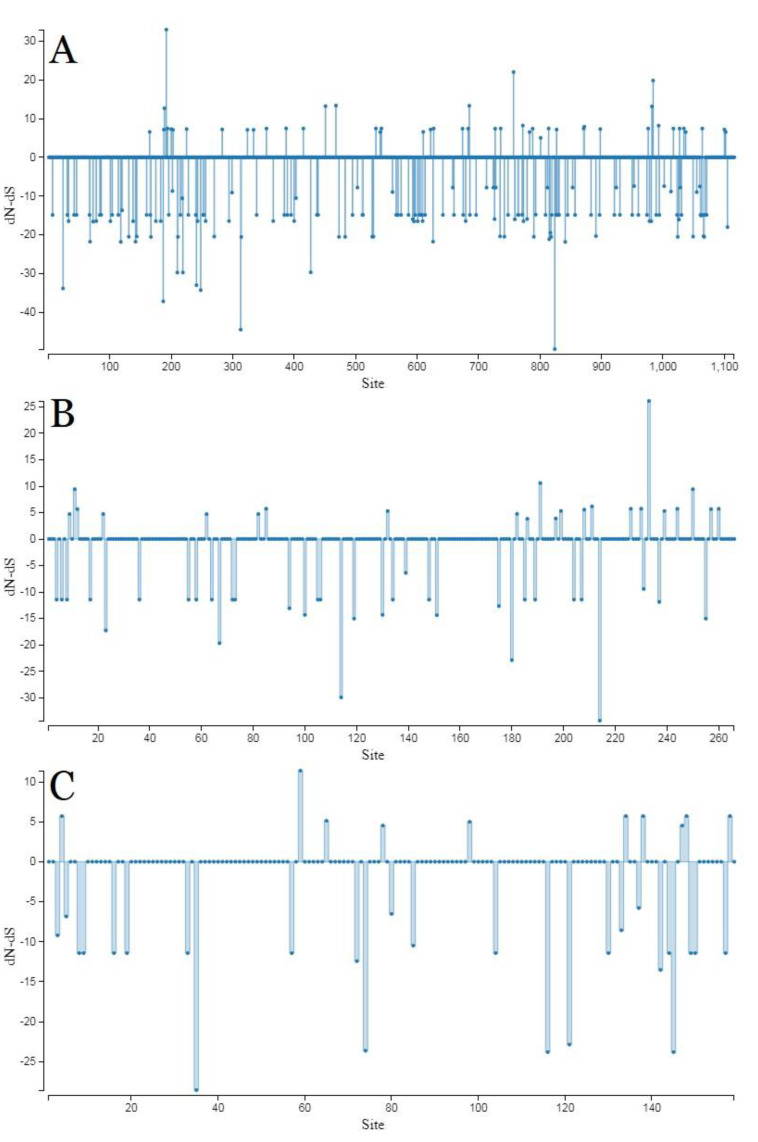
SLAC site graph to identify positive and negative codons/sites for ORF2 (**A**), ORF3 (**B**), and ORF4 (**C**).

The ToBRFV genome was under strong purifying pressure, as shown by the low dN/dS ratio (ω < 0.2) assigned to all of the analysed ORFs and geographic regions. Group 2 and “Middle East+China” populations consistently obtained significantly higher ω values than other populations in both comparisons based on the complete genome and CP sequences, in the phylogroup and region categories, respectively (Table 1). On the basis of the ORFs (ORF2, ORF3, ORF4), there were no codons under positive selection by the SLAC method in HyPhy software package, which was implemented in Datamonkey webserver with a *p*-value threshold (*p* ≤ 0.1) (Figure 3). These data showed that ToBRFV was under strong negative evolutionary constraints on the ORFs.

### 2.4. Neutrality Tests

Neutrality tests using three parameters consistently gave negative values to all tested populations in the analysis of the complete genome, four ORFs, and geographic regions. All tests on the different genome regions obtained significant values and indicated there were enough data to support the statistical calculations (Table 2).

### 2.5. Gene Flow and Genetic Separation among Populations

Gene flow study by DnaSP assigned high *K*_S_*, *K*_ST_*, Z*, and *S*_nn_ metrics, with a significant *p*-value in comparisons between groups 1 and 2, in both analyses of the complete genome and CP sequences, indicated that the clustering of these groups had been conducted correctly. Additionally, in both analyses based on the complete genome and CP sequences, *F*_ST_ values for group 1 vs. 2 were estimated to be > 0.25, whereas for all isolates vs. group 2 were < 0.25 (0.0281 and 0.0748, respectively) (Table 3).

In the region category, all comparisons obtained relatively low *K*_S_*, *K*_ST_*, Z*, and *S*_nn_ metrics with significant and non-significant *p*-values. Additionally, *F*_ST_ values < 0.25, which indicated frequent gene flow and no genetic differentiation over time, were also assigned to all comparisons based on the complete genome and CP sequences (Table 3).

### 2.6. Molecular Dating

**Figure 4:**
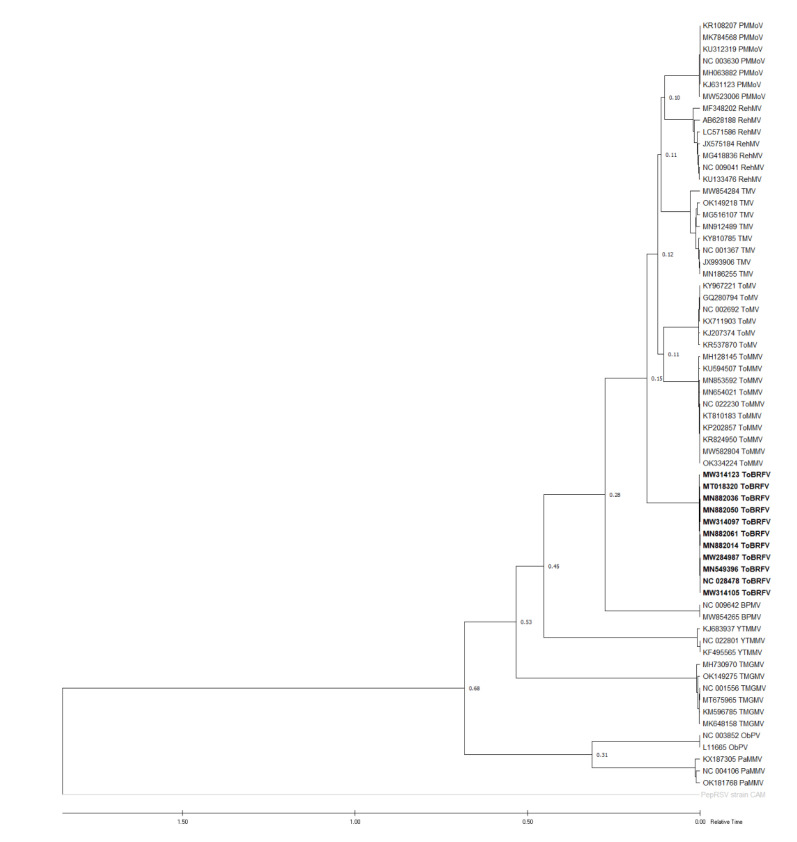
Divergence time estimation of 11 tobamoviruses with Asterids as main hosts: pepper mild mottle virus (PMMoV), rehmannia mosaic virus (RheMV), tobacco mosaic virus (TMV), tomato mosaic virus (ToMV), tomato mottle mosaic virus (ToMMV), tomato brown rugose fruit virus (ToBRFV), bell pepper mottle virus (BPMV), yellow tailflower mild mottle virus (YTMMV), tobacco mild green mosaic virus (TMGMV), obuda pepper virus (ObPV), and paprika mild mottle virus (PaMMV) by RelTime-ML in MEGA X. An isolate of pepper ringspot virus (PepRSV, genus *Tobravirus*) was used as out-group.

Eleven ToBRFV isolates from different phylogroups: nine, group 1; one, group 2; and one, group 3, were compared with ten other tobamovirus isolates from various countries and hosts available in GenBank. The constructed ML showed that a subgroup consisting of ToBRFV, ToMMV, ToMV, TMV, RehMV, and PMMoV populations was split with the BPMV population at around the middle time over the course of the evolution of 11 tested tobamoviruses (Figure 4). Similarly, percentage identity analysis by SDT v1.2 deduced that ToBRFV shared higher nt and aa identities with ToMMV, ToMV, TMV, RehMV, and BPMV than other compared tobamoviruses (Table 4). Because there is no available report on MRCA based on the complete genome sequences of tobamoviruses, the divergence time of ToBRFV was calculated using the ratios of ToBRFV and other patristic distances. Using this method, the divergence time of ToBRFV with BPMV was estimated to be 1.87 and the divergence time with the ToMMV, ToMV, TMV, RehMV, and PMMoV groups was estimated to be 0.8. TMRCAs of other tobamoviruses with ToBRFV are presented in Table 5.

## 3. Discussion

The naturally stable and easily mechanically transmitted tobamoviruses have always been serious problems to tomato, pepper, and other *Solanaceae* cultivation. Therefore, cultivars carrying the resistance genes *Tm-1*, *Tm-2*, and *Tm-2^2^* became the most effective measures to control members of the genus, such as ToMV and TMV [30,31,32]. ToBRFV then came along relatively recently and broke all those widely implemented highly resistant (HR) genes and was poised to become a dominant species [9,33,34]. However, the tolerance of some genotypes of *Solanum pimpinellifolium* [35] and the resistance of *Solanum ochrantum* [36] to the virus had been demonstrated. The knowledge on the genetic diversity and population structure of ToBRFV reported in the current study could also contribute to further the biological arms race to identify potential HR genes and their longevity.

With a low disease incidence of 3.6%, ToBRFV was shown to have limited spread to other regions after its initial detection in Southern Turkey [14]. The appearance of spots on fruits (Figure 1) seems to be due to discolouration only, different than those caused by tomato spotted wilt virus (TSWV, genus *Orthotospovirus*), which mostly have dark halos around the discoloured areas. In addition to that, TSWV infections usually do not lead to severe blisters on fruits [37]. Tomato and pepper plants in greenhouses where ToBRFV was found were promptly eradicated and the greenhouses and tools were thoroughly sterilised using disinfectant. These greenhouses were barred from planting anything for one year. 

When full genome sequence data were absent, the phylogenetic relationships among the isolates of tobamoviruses were often established using full CP sequences [38,39,40]. Therefore, the three groups of ToBRFV phylogeny, based on 480 bp full CP gene nt sequences, were confirmed in this study. The clustering of the 49 isolates of group 1 survived phylogenetic analyses on the complete genome, and three other ORFs indicated a single recent common ancestor for all group 1 isolates. Meanwhile, the members of group 2 were dispersed into different groups in four other trees. Isolate no. MW284987, which belongs to group 3 in the CP tree, was always positioned among the members of group 2 in four other trees. 

Phylogenetic analysis on the complete CP gene sequence positioned all seven novel isolates in group 2, indicating high identity among these isolates even with the TR-79 (OM810274) isolate, which was from pepper. Further observations also did not find an association between isolates clustering with the host species or origin. Interestingly, one of the previously registered Turkish isolates, TBRFV-Ant-Tom (MT107885), formed a monophyletic subcluster separated from all novel isolates, suggesting multiple recent intrusions into the country.

In line with phylogenetic analysis, a genetic variation study using DnaSP software also determined high identity among current global ToBRFV isolates. The CP gene was deduced as the most conserved region with the lowest genetic variation according to all parameters, thereby supporting the suitability of CP sequences to help to resolve ToBRFV phylogeny.

Vigorous purifying selections were observed in the complete genome and all four ORF sequences (ω = 0.0869–0.2066). Due to the SLAC result, all of the codons/sites were under negative selection, whereby it seems probable that negative sites have an important role in infection intensity and virus transmission. In CP, group 1 isolates were under more intense negative evolutionary constraints compared to isolates in group 2. However, group 1, together with group 2 and all regional populations, were all shown to be under weak purifying selections and assigned negative values by neutrality tests. Therefore, these populations are experiencing expanding or bottleneck selections, with the growth likely due to low-frequency polymorphism (new mutations); thus, further division into subgroups or even new groups can be anticipated from these populations.

The high *K*_S_*, *K*_ST_*, Z*, and *S*_nn_ values, supported by significant *p*-values, showed that there is a large genetic variation among group 1 and group 2. In line with this, *F*_ST_ values for group 1 vs. 2, in both the complete genome and CP, were consistently more than 0.25 (0.3987 and 0.6758, respectively), indicating strong genetic separation between them. Therefore, these results corroborated the results of phylogenetic analysis.

Tomato and pepper have been cultivated for thousands of years in their native South America and for hundreds of years in other regions since their introduction. It is interesting that such an evolutionarily successful ToBRFV, of which natural hosts are mostly restricted to two plant species, has just been detected recently, much later than other important tobamoviruses infecting *Solanaceae*. Therefore, its origin and genetic relation with the other members of *Tobamovirus* become necessary to understand.

So far, South America is thought to be the origin of the virus, mainly due to the position of one Peruvian isolate, 36783571_2 (MW314111), in a phylogenetic tree based on complete genome sequences [8]. Likewise, the complete genome tree constructed in the current study also positioned 36783571_2 in an early diverging cluster, together with three isolates from Peru: 40732089_3 (OM515235), 41108421 (OM515258), and 36364500_1 (OM515233), and four isolates from Netherlands: 39563433_3 (MW314123), 39563361_A (MN882042), 39563361_B (MN882043), and 41903353 (OM515264) (Figure 2). This showed that the more complete genome sequences of isolates from South America and other regions are clearly needed to complement the already abundant European isolates in order to resolve the origin of ToBRFV, as also stated by [8]. In line with genetic variation and gene flow analyses, a quantitative measurement using Fixation index also concluded high identity among global isolates, and that there is no clear genetic separation among isolates from different origins at present, as demonstrated by *F*_ST_ values between three regions: Europe, “Middle East+China”, and America were all 0.25 or less (Table 3).

Analysis on CP nt sequences estimated that the divergence between ribgrass mosaic virus (RMV) and a subgroup that consists of PMMoV, TMGMV, ToMV, and TMV happened 1987–5268 years ago [41]. Another molecular dating analysis based on replicase gene sequences showed that the group contains BPMV, ToMV, TMV, RehMV, and PMMoV, which diverged from another group that consists of PaMMV, ObPV, TMGMV along the tobamovirus evolution timeline [42], very similar to analysis based on the complete genome by [43] and this current study. However, the estimated TMRCAs of tobamoviruses based on the complete genome sequences comparison were not available. Therefore, a simple estimation of the TMRCA of ToBRFV was conducted using the ratios of the virus patristic distance with other tested tobamoviruses provided by MEGA X software. TMRCA of ToBRFV was determined to be the closest with BPMV, with a ratio of 1.87. The constructed TimeTree pictured ToBRFV emergence as a distinct species was not recent, around the middle of the course of genus *Tobamovirus* evolution (Figure 4).

## 4. Materials and Methods

### 4.1. Samples Collection

Surveys were conducted to detect the possible spread of ToBRFV into greenhouses located in Ankara (A), Eskişehir (E), Bartın (B), and Zonguldak (Z) provinces, which represented the centre, northwest, and north regions of Turkey, respectively. Leaf samples were taken from 195 plants: 112 tomato (A 37, E 20, B 42, and Z 13) and 83 pepper (A 14, E 26, B 7, and Z 36), showing viral symptoms such as leaf mosaic, leaf yellowing, spots on fruits, blisters on fruits, plant malformation, and stunting. An individual sample was put into a plastic bag, labelled, then kept at −20 °C until further use.

### 4.2. Serological Test

The collected samples were tested for ToBRFV infection using the double-antibody sandwich enzyme-linked immunosorbent assay (DAS-ELISA) technique as described by the manufacturer (Loewe, Germany). A Multiscan FC Microplate Photometer plate reader (Thermo Scientific, USA) was used to evaluate colour development in the plates at a wavelength of 405 nm. A sample was considered positive if its average absorbance value was at least twice that of the negative control.

### 4.3. RNA Isolation, RT-PCR, and Sequencing

Total RNA was extracted from leaves of all collected samples using the “NucleoSpin RNA Plant Mini kit” according to the manufacturer’s protocol (Macherey-Nagel, Duren, Germany). The concentration and purity of the obtained total RNA were determined with the help of a spectrophotometer (NanoDrop 2000, ThermoFisher Scientific, Waltham, MA, USA); total RNA concentrations of all samples were diluted to 50 ng.

The 25 μL one-step RT-PCR reaction mixture was composed of 6 μL 5X Go Taq Flexi Green buffer, 1.2 μL 25 mM MgCl_2_, 0.7 μL 10 mM dNTPs, 1 μL each of 10 μM forward and reverse primer, 0.1 μL Reverse Transcriptase (ProtoScript^®^ II Reverse Transcriptase-M-MuLV), 0.1 μL RNase (RNase Inhibitor, Murine 1 U/μL), 0.25 μL Taq DNA Polymerase (GoTaq^®^ G2 Flexi DNA Polymerase 5 U/μL), and 2 μL of 50 ng/μL total RNA. ToBRFV-F (5503) and ToBRFV-R (6344) primer pairs [10] to amplify 842 bp of partial MP (209 bp), complete CP (480 bp), and 3′-UTR (153 bp) regions were applied in the PCR step. An RT-PCR amplification program of 20 min at 42 °C, 5 min at 94 °C, 30 cycles of 60 s at 94 °C, 60 s at 48 °C, and 60 s at 72 °C, followed by a final extension for 10 min at 72 °C, was performed using a thermal cycler (Biometra, Analytik Jena, Germany).

RT-PCR products were mixed with loading buffer (ThermoFisher Scientific, Waltham, MA, USA) (7:1), then loaded into ready-to-use agarose gel stained with Pronosafe DNA fluorescent marking (Condalab, Madrid, Spain), and then run in an electrophoresis apparatus (ThermoFisher Scientific, Waltham, MA, USA) at 100 V for 1 h. The appearance of the targeted specific band was observed under a UV transilluminator (Vilber, Marne-la-Vallée, France). The amplicons of positive tested samples were TA cloned in pGEM-T Easy vector (Promega Corp., Madison, WI, USA), and then shipped to a commercial firm (BM Laboratory Systems, Ankara, Turkey) for bidirectional Sanger sequencing.

### 4.4. Recombination Analysis and Phylogeny

In September 2022, all 174 isolates with the complete genome sequences available in NCBI GenBank were retrieved and aligned together using the ClustalW algorithm implemented in MEGA X v.10.2.4 program [44]. The 5′ and 3′-UTR regions were trimmed to create the “complete genome” alignment (6117 bp). Separately, to assess the phylogeny and genetic structure of each ORF, alignment was also trimmed according to ORF1/small replicase (3351 bp), ORF2/RdRp (1422 bp), ORF3/MP (801 bp), and ORF4/CP (480 bp) sequences, respectively. The complete CP sequences of four other GenBank isolates and also novel isolates obtained in this study were added to ORF4/CP alignment (Table S1).

The presence of any phylogenetic anomaly in the alignments was scanned using Recombination Detection Program 4 (RDP4) with its full suite of options: RDP, Chimaera, MaxChi, Bootscan, Siscan, GENECONV, and 3Seq, in default parameters [45]. Anomalies detected by fewer than five algorithms with a Bonferroni-corrected *p*-value of < 0.05 were ignored.

The best substitution model to construct a Neighbor-Joining (NJ) phylogenetic tree based on each alignment was assessed by MEGA X to be T92 [46] plus uniform rates among sites, with 1000 bootstrap replicates. The percentage identities of the nucleotide (nt) and amino acid (aa) sequences of the complete genome of ToBRFV were calculated using Sequence Demarcation Tool (SDT) v1.2 software [47].

### 4.5. Population Structure of ToBRFV

The number of haplotypes (h), haplotype diversity (Hd), the number of variable sites (*S*), the total number of mutations (η), the average number of nt differences between sequences (*k*), and nt diversity (per site) (π) were analysed using the DnaSP v.6.12.03 program [48] to determine genetic variation in the complete genome, ORF1, ORF2, ORF3 (MP), and ORF 4 (CP) sequences of different populations. Additionally, transcriptional selection (dN/dS ratio = ω) was also estimated. When ω is > 1, equal = 1, and < 1, the related genome region is evaluated to be under positive (diversifying), neutral, or negative (purifying) selection, respectively. To evaluate the dS/dN, individual codon positions for each ORF (ORF1, ORF2, ORF3, ORF4) under natural selection were used for single likelihood ancestor counting algorithm (SLAC) (implemented in MEGA X software and available free online on Datamonkey webserver [49].

To calculate the neutrality, Fu and Li’s D*, Fu and Li’s F* [50], and Tajima’s *D* [51] metrics were used with a window length of 100 sites and a step size of 25 sites. The genetic differentiation and gene flow among populations were assessed using the *K*_S_*, *K*_ST_*, Z*, *S*_nn_, and *F*_ST_ (fixation index) [52]. For panmictic populations, the *F*_ST_ value was 0, while a ratio higher than 0.25 implies expanding genetic separation [53,54]. 

### 4.6. Molecular Dating Analysis

Lineages of tobamoviruses were determined to be congruent with those of their hosts [43]. Therefore, the divergence times of the ToBRFV population with 10 other Asterids-infecting tobamoviruses that are known to have close genetic relationship [43]: pepper mild mottle virus (PMMoV), rehmannia mosaic virus (RheMV), tobacco mosaic virus (TMV), tomato mosaic virus (ToMV), tomato mottle mosaic virus (ToMMV), bell pepper mottle virus (BPMV), yellow tailflower mild mottle virus (YTMMV), tobacco mild green mosaic virus (TMGMV), obuda pepper virus (ObPV), paprika mild mottle virus (PaMMV), and a *Tobravirus* species: pepper ringspot virus (PepRSV) as outgroup, were estimated based on the age evaluation of internal nodes [44]. TimeTree was reconstructed using the fast-dating RelTime-ML computational method under the Tamura 3-parameter model (Tamura, 1992) implemented in MEGA X software, with a default calibration of TMRCA (time to most recent common ancestors) [55].

## 5. Conclusions

The clustering of ToBRFV isolates into three phylogroups was confirmed. Currently known isolates were mostly sampled from Europe, and some were from the Middle East; thus, there was a very high genetic identity among them. On the basis of the few available data, the South American isolates, when sampled more in the future, may provide more variation and explain better the origin and ancestor of the global ToBRFV population.

## Figures and Tables

**Table 1:** Genetic diversity and polymorphism analyses of the complete genome, ORF1, ORF2, ORF3 (MP), and ORF4 (CP) of ToBRFV from different phylogroups and regions.

Population	N	h	Hd	*S*	η	*k*	π	dS	dN	ω
**Complete genome**	174	123	0.994	377	390	17.396	0.0028	0.0088	0.0011	0.125
**Phylogroup**										
Group 1	49	35	0.985	62	63	5.639	0.0009	0.0029	0.0004	0.1379
Group 2	124	87	0.991	326	333	17.719	0.0029	0.0087	0.0012	0.1379
**Region** ^1^										
Europe	130	86	0.991	235	241	17.372	0.0028	0.0089	0.0011	0.1236
ME and China	34	28	0.988	132	134	12.882	0.0021	0.0059	0.0009	0.1525
America	10	10	1.000	49	50	14.667	0.0024	0.0081	0.0008	0.0988
**ORF1**	174	112	0.989	283	292	12.789	0.0026	0.0085	0.0009	0.1059
**ORF2**	174	103	0.984	204	205	8.236	0.0025	0.0081	0.0008	0.0988
**ORF3 (MP)**	174	52	0.933	60	63	3.674	0.0046	0.0121	0.0025	0.2066
**ORF4 (CP)**	185	34	0.646	39	40	0.994	0.0021	0.0069	0.0006	0.0869
**Phylogroup**										
Group 1	49	7	0.267	7	7	0.325	0.0007	0.0025	0.0001	0.04
Group 2	134	25	0. 421	29	29	0.637	0.0013	0.0035	0.0007	0.2
**Region** ^1^										
Europe	131	21	0.668	23	24	0.987	0.0021	0.0072	0.0005	0.0694
ME and China	42	10	0.458	12	12	0.707	0.0015	0.0033	0.0009	0.2727
America	12	5	0.576	7	7	1.167	0.0024	0.0088	0.0005	0.0568

N: number of isolates, h: number of haplotypes, Hd: haplotype diversity, S: number of variable sites, η: total number of mutations, k: average number of nucleotide differences between sequences, π: nucleotide diversity (per site), dN: non-synonymous nucleotide diversity, dS: synonymous nucleotide diversity, and ω: dN/dS. ^1^ Isolate origin—**Europe**: Belgium, France, Germany, Greece, Italy, Netherlands, and UK; **Middle East and China**: Cyprus, Egypt, Iran, Israel, Jordan, Palestine, Turkey, and China; and **America**: Canada, Mexico, Peru, and USA.

**Table 2:** Results from demography test statistics between sequences of the complete genome, ORF1, ORF2, ORF3 (MP), and ORF4 (CP) of ToBRFV populations.

Population	Fu and Li’s *D* *	Fu and Li’s *F* *	Tajima’s *D*
**Complete genome**	−5.56444 **	−4.81135 **	−2.40531 **
**Phylogroup**			
Group 1	−2.18556 ns	−2.57257 *	−2.10472 *
Group 2	−4.89980 **	−4.48389 **	−2.36401 **
**Region** ^1^			
Europe	−3.60441 **	−3.43963 **	−1.99775 *
ME and China	−2.48265 ns	−2.86841 *	−2.28997 **
America	−1.10835 ns	−1.17368 ns	−0.83168 ns
**ORF1**	−5.33498 **	−4.69009 **	−2.41119 **
**ORF2**	−5.63445 **	−4.93305 **	−2.46251 **
**ORF3 (MP)**	−3.27960 **	−3.27892 **	−2.03581 *
**ORF4 (CP)**	−6.29855 **	−5.63807 **	−2.51446 ***
**Phylogroup**			
Group 1	−3.41067 **	−3.51604 **	−2.10661 *
Group 2	−5.37507 **	−5.10880 **	−2.58167 ***
**Region** ^1^			
Europe	−4.81828 **	−4.56208 **	−2.23084 **
ME and China	−3.17348 *	−3.39447 **	−2.27783 **
America	−2.38877 *	−2.57986 *	−1.94368 *

* 0.01 < *p*-value > 0.05; ** 0.001 < *p*-value > 0.01; *** *p*-value < 0.001; and ns: not significant. ^1^ Isolate origin—**Europe**: Belgium, France, Germany, Greece, Italy, Netherlands, and UK; **Middle East and China**: Egypt, Iran, Israel, Jordan, Palestine, Turkey, and China; and **America**: Canada, Mexico, Peru, and USA.

**Table 3:** Genetic differentiation estimates for lineages of ToBRFV, based on the complete genome and CP gene sequences comparison.

Comparison	^α^*K*_S_*	^α^*K*_ST_ *	*p*-Value	^α^*Z* *	*p*-Value	*S*_nn_	*p*-Value	^β^ *F*_ST_
**Complete genome**								
**Phylogroup**								
All (*n* = 174)/Group 1(*n* = 49)	2.5781	0.0421	0.0000 ***	8.9495	0.0000 ***	0.6405	0.7180 ns	0.2551
All (*n* = 174)/Group 2 (*n* = 124)	2.7892	0.0066	0.0000 ***	9.6674	0.0000 ***	0.3144	1.0000 ns	0.0281
Group 1 (*n* = 49)/Group 2 (*n* = 124)	2.5164	0.0988	0.0000 ***	8.1317	0.0000 ***	1.0000	0.0000 ***	0.3987
**Region** ^1^								
Europe (*n* = 130)/ME and China (*n* = 34)	2.6901	0.0279	0.0000 ***	8.3888	0.0000 ***	0.9415	0.0000 ***	0.1291
Europe (*n* = 130)/America (*n* = 10)	2.7292	0.0151	0.0000 ***	8.1349	0.0000 ***	0.9929	0.0000 ***	0.1836
ME and China (*n* = 34)/America (*n* = 10)	2.5216	0.0295	0.0000 ***	5.7025	0.0000 ***	0.9886	0.0000 ***	0.1293
**ORF4 (CP)**								
**Phylogroup**								
All (*n* = 185)/Group 1 (*n* = 49)	0.4993	0.1687	0.0000 ***	9.1645	0.0000 ***	0.7642	0.0000 ***	0.4498
All (*n* = 185)/Group 2 (*n* = 134)	0.4899	0.0369	0.0000 ***	9.9451	0.0000 ***	0.5081	0.6070 ns	0.0748
Group 1 (*n* = 49)/Group 2 (*n* = 134)	0.3298	0.4091	0.0000 ***	8.5045	0.0000 ***	1.0000	0.0000 ***	0.6758
**Region** ^1^								
Europe (*n* = 131)/ME and China (*n* = 42)	0.5396	0.0494	0.0000 ***	8.6861	0.0000 ***	0.7149	0.0000 ***	0.1451
Europe (*n* = 131)/America (*n* = 12)	0.5807	0.0175	0.0120 *	8.3262	0.0220 *	0.8674	0.0210 *	0.1124
ME and China (*n* = 42)/America (*n* = 12)	0.4511	0.0065	0.2500 ns	6.4269	0,2870 ns	0.6580	0.3070 ns	−0.0005

* 0.01 < *p*-value > 0.05 and *** *p*-value < 0.001. ^α^ *K*_S_*, *K*_ST_*, *Z*, and *S*_nn_ are test statistics of genetic differentiation; ^β^ *F*_ST_ is a coefficient of gene differentiation, which measures inter-population diversity; ns: not significant. ^1^ Isolate origin—**Europe**: Belgium, France, Germany, Greece, Italy, Netherlands, and UK; **Middle East and China**: Egypt, Iran, Israel, Jordan, Palestine, Turkey, and China; and **America**: Canada, Mexico, Peru, and USA.

**Table 4:** Identity percentage of the complete genome nucleotide and amino acid sequences of ToBRFV with other Asterids-infecting tobamoviruses.

ToBRFV vs.	Identity (%)	
	Nucleotide	Amino Acid
PMMoV	69–69.6	61.8–62.3
RehMV	80.1–81.5	84–85.9
TMV	81.2–81.7	85.3–86.7
ToMV	81–81.4	85.5–86.4
ToMMV	80.7–81–1	84.4–85.5
BPMV	75.4–76.1	71.4–72.1
YTMMV	64.1–64.5	57.9–58.4
TMGMV	65.1–65.5	62.5–63.1
ObPV	65–65.1	62.9–64
PaMMV	65.3–65.7	63.7–64.1

**Table 5:** Estimated ToBRFV TMRCA based on the ratio of the patristic distances within the ten Asterids-infecting tobamoviruses’ complete genome sequence maximum likelihood tree.

Species	Mean Patristic Distance	Ratios of ToBRFV and Other Patristic Distances
PaMMV	0.68	4.53
ObPV	0.68	4.53
TMGMV	0.53	3.53
YTMMV	0.45	3
BPMV	0.28	1.87
ToBRFV	0.15	1
ToMMV	0.12	0.8
ToMV	0.12	0.8
TMV	0.11	0.73
RehMV	0.10	0.67
PMMoV	0.10	0.67

## Data Availability

Partial genome sequences of seven novel Turkish ToBRFV isolates are available in GenBank, reference number OM810270, OM810271, OM810273, OM810274, OM810277, OM810278, and OM810279.

## Supplementary Materials

The following supporting information can be downloaded at: https://www.mdpi.com/article/10.3390/plants11233279/s1, Table S1: Tomato brown rugose fruit virus (ToBRFV) isolates tested in this study.
